# Management Strategies for Failed Pilon Fractures: A Personalized Approach to Revision Reconstruction

**DOI:** 10.3390/jpm15120602

**Published:** 2025-12-05

**Authors:** Lauren Luther, Richard S. Moore III, Sriranjani Darbha, Bethany Gallagher, Daniel J. Stinner

**Affiliations:** 1 Department of Orthopedic Surgery, Vanderbilt University Medical Center, 1215 21st Ave. South, Suite 4200, MCE, Nashville, TN 37232, USA; 2 Texas A&M College of Medicine, Houston, TX 77030, USA

**Keywords:** pilon, revision surgery, nonunion, malunion, tibiotalar arthrodesis, total ankle arthroplasty, bone defect reconstruction, limb salvage, tibiotalar arthritis, post-traumatic arthritis, personalized medicine, fracture related infection

## Abstract

Despite advances in staged protocols and fixation techniques, treatment of pilon fractures remains a significant challenge in orthopedic trauma, with up to 21% of patients requiring revision surgery. Management of a pilon fracture that has failed initial treatment involves navigating a myriad of complicating variables, including infection, bone loss, malalignment, and nonunion. Although no single surgical approach can be aptly applied to the broad range of pathology and severity spanned by these patients, this narrative review provides a systematic framework for developing a revision pilon reconstruction plan. We present a protocol for pre-operative assessment and review current techniques for infection eradication, bone defect management, deformity correction, and joint-preserving versus joint-sparing surgery. These fundamental strategies form the foundation of a successful salvage plan and can be personalized to address specific fracture morphology, host factors, and goals of care.

## 1. Introduction

Despite advances in implants and surgical technique, management of pilon fractures remains a challenge within orthopedics. These complex injuries of the tibial plafond most commonly result from high-energy mechanisms delivering an axial load to the lower extremity, driving the talus into the distal tibia [1]. The resulting fracture morphology is characterized by impaction of the articular surface and metaphyseal comminution [1]. The osseous damage is accompanied by substantial disruption of the minimal soft tissue envelope surrounding the distal tibia [2]. Open fractures are common in this anatomic area, but even closed fractures are associated with profound compromise of the local healing environment [3].

Unsurprisingly, in light of these significant challenges to reduction, fixation, and union, complications following operative fixation of pilon fractures are common. The development of staged protocols and surgical techniques emphasizing respect for soft tissues has improved outcomes, but recent series still report complications requiring revision in up to 21.2% of cases [3,4,5]. Even when compared to other high-energy lower extremity traumatic injuries, pilon fractures have worse outcomes and poorer long-term function [5,6]. Given the frequency of failed initial fixation, strategies for revision pilon reconstruction are an important component of an orthopedic traumatologist’s skillset.

The complexity and variety of pilon fractures make each revision case a unique challenge. The optimal treatment strategy requires a personalized approach, tailored to both the injury and the host [7,8]. However, there are common themes of failure that need to be navigated in the revision pilon setting—infection, bone defects, malunion, nonunion, and symptomatic post-traumatic arthritis [9]. Patients often present with a constellation of these problems that need to be addressed in concert [10].

The purpose of this narrative review is to provide a reproducible, evidence-based framework that can be tailored to address the wide spectrum of pathology comprised by pilon fractures requiring revision. We define the failed pilon fracture as one that develops infection, nonunion, malunion, or symptomatic post-traumatic arthritis after an initial attempt at operative fixation. The review addresses pre-operative planning considerations for the failed pilon and then provides an overview of tactics for addressing infection, bone loss, and deformity. We conclude by discussing joint-sparing and joint-sacrificing revision surgical options.

## 2. Methods

### Search Strategy and Selection Criteria

The PubMed database was searched for English-language articles published between 2000 and 2025 using two domains of medical subject headings (MeSH) terms and keywords combined with “AND”, and within the two domains, the terms were combined with “OR.” The first domain included words related to pilon fractures (“pilon fracture”, “tibial plafond fracture”, “distal tibia fracture”), and the second domain included words related to revision surgery (“revision”, “reconstruction”, “salvage”, “arthrodesis”, “fusion”, “osteotomy”, “infection”, “nonunion”, “malunion”). Titles and abstracts were independently screened by two reviewers (L.L., S.D.). Articles focused on primary management of pilon fractures, reconstruction after oncologic resection, management of rotational ankle fractures, or pediatric patients were excluded. The remaining articles were grouped thematically according to common complications encountered in the management of failed pilon fractures—infection, bone loss, malunion, and post-traumatic arthritis. Relevant clinical examples from the senior authors’ practice (D.J.S., B.G.) were also included.

## 3. Pre-Operative Assessment of Failed Pilon

The approach to a symptomatic pilon fracture nonunion or malunion should begin with a thorough clinical and operative history. Important components of the physical exam include noting the location of prior skin incisions and assessing for foot deformities that may have developed as compensatory mechanisms for malalignment or secondary to prolonged periods of non-weight bearing [9]. If flap coverage was previously required, a thorough understanding of the pedicle path is imperative to avoid disrupting it during revision [9]. Full-length weight-bearing radiographs of the bilateral lower extremities and hindfoot alignment radiographs should be obtained to analyze overall limb posture and identify potential sources of deformity [11,12]. Computed tomography (CT) plays a critical role in assessing the extent of fracture healing and characterizing intra-articular pathology [11]. Three-dimensional reconstruction software is increasingly used for pre-operative planning in the setting of primary pilon fixation and can also be beneficial in the revision setting for evaluating the deformity morphology and planning osteotomies [13,14]. Adequate vascularity to support bone healing should be confirmed [10]. Standard non-union labs assessing for infection and impaired bone metabolism should be collected. Biomarkers associated with fracture healing are an active area of research and may provide novel markers for nonunion in the future [15]. Of note, infectious laboratory markers within the normal range do not rule out infection, and in the setting of elevated clinical suspicion for infection, biopsy remains the gold standard and should be considered prior to undertaking a larger reconstruction procedure. Pre-operative optimization and post-operative recovery benefit from multidisciplinary collaboration [11]. Depending on the pathology, plastic surgery, vascular surgery, infectious disease, and physical therapy experts can have a profound impact on a patient’s outcomes and should be involved early in the planning process when indicated [9,10,16]. Modifiable risk factors for further complications should be addressed if possible, including malnutrition, nicotine use, and untreated mental health or endocrine disorders [8,17,18,19,20]. Defining the scope of the problem and mobilizing appropriate resources lays the foundation for the development of a personalized reconstruction plan.

## 4. Strategies to Address Common Challenges in Revision Pilon Reconstruction

### 4.1. Infection

Eradication and prevention of infection are of paramount importance when attempting revision of a pilon fracture. The distal tibia is surrounded by a relatively thin soft tissue envelope, predisposing this area to open fractures at the time of initial injury. Due to the high-energy mechanisms implicated in pilon fractures, even closed fractures result in significant trauma to the soft tissue envelope [2]. Although complication rates following operative fixation of pilon fractures have improved with the development of staged protocols and emphasis on surgical technique that respects soft tissues, infection remains a significant problem [1]. A modern retrospective series at a center employing a staged protocol found an infection rate of 23.2% in open fractures and 11.3% in closed injuries [16].

The cornerstone of infection management is adequate debridement [9]. Definitely differentiating viable and nonviable tissue during debridement is an unsolved problem, although fluorescence-guided technologies have shown promise in early studies [21]. Until more sophisticated debridement tools are available, it is recommended to err on the side of radical debridement and excise any questionably nonviable tissue [9,10]. A variety of strategies exist for addressing bony and soft tissue defects in an aseptic environment, whereas proceeding with definitive reconstruction in an infected wound bed can result in a recalcitrant problem and potentially amputation. The success of subsequent efforts to restore length, alignment, and function, as well as the efficacy of the techniques described in this review to accomplish those goals, is predicated on the elimination of infection.

Local and systemic antibiotic therapy augments a thorough debridement of necrotic and infected tissue [9,22]. Antibiotic therapy should be managed in collaboration with infectious disease experts and driven by intra-operative culture [16]. Polymerase chain reaction testing may help further customize antibiotics to individual patients in the future by allowing for rapid species identification and sensitivity testing [23]. Antibiotic powders, antibiotic beads, and antibiotic-impregnated calcium sulfate or polymethyl methacrylate (PMMA) are options for creating higher local concentrations of antibiotics than can be achieved with systemic therapy [4,9,10,24]. In addition to serving as vehicles for antibiotic delivery, calcium sulfate and PMMA can act as temporary deadspace management until the recipient bed is suitable for bone grafting [25].

The majority of deep infections following a pilon fracture present prior to full fracture healing [16]. Infected nonunion presents a more challenging problem than isolated infection because maintaining existing implants may result in retained colonies of bacteria, but removal results in instability. Instability can cause persistent inflammation, which can serve as a barrier to both infection clearance and fracture healing [26]. External fixation provides adequate stability without requiring the placement of foreign material within the infected field. Half-pin external fixation constructs can be utilized as part of a staged protocol with planned definitive fixation after resolution of infection [9]. Tensioned thin-wire circular external fixation (TWCEF) is a more sophisticated and complex option that can be placed during initial debridement and also utilized for definitive fixation [10]. Less commonly, in patients who are poor candidates for external fixation or refuse external fixation, an antibiotic-coated intramedullary device can be used [24]. The antibiotic coating aims to minimize bacterial biofilm formation and thereby decrease the risk of creating a nidus for persistent infection. Although initially developed for diaphyseal infected nonunions, the technique has also been applied to tibiotalocalcaneal nails for infected pilon nonunions [27].

### 4.2. Bone Defects

**Figure 1:**
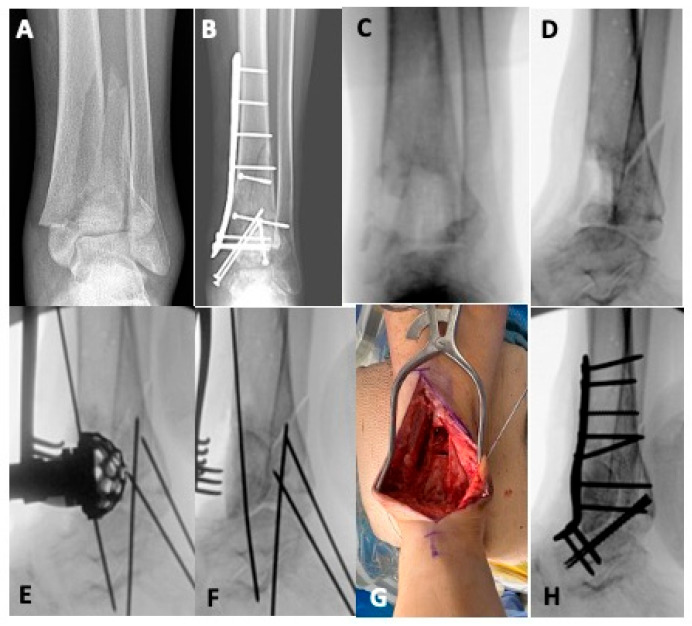
A twenty-four-year-old sustained a pilon fracture in a motor vehicle collision. Initial radiographs (**A**) demonstrated an AO 43-C fracture with metaphyseal comminution. His course was complicated by infection, and despite multiple attempts at irrigation and debridement with hardware retention at an outside facility. He presented to our institution with an infected nonunion and draining sinus (**B**) at six months after his original injury. He was managed in a staged fashion. After removal of hardware and adequate debridement of nonviable bone and prior bone graft, he had a large anterior defect (**C**,**D**) that was temporized with antibiotic-impregnated cement. After infection clearance, he was managed definitively with tibiotalar arthrodesis using autologous cancellous bone graft and structural femoral head allograft to fill the bone void. An acetabular reamer was used to prepare the graft bed (**E**), then a femoral head allograft was appropriately fashioned to fill the defect (**F**,**G**), followed by a compression plate and screw construct for tibiotalar arthrodesis (**H**).

**Figure 2:**
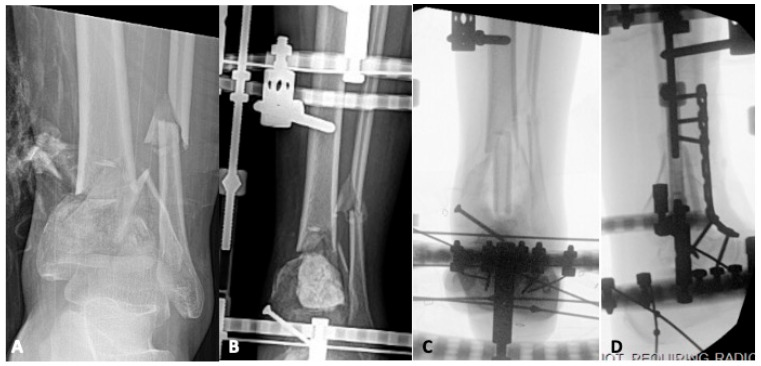
A twenty-six-year-old who sustained a Gustilo-Anderson Type IIIb pilon fracture in a motor vehicle collision. Her initial injury (**A**) was characterized by bone loss and comminution and her course was complicated by an infected nonunion. (**B**) The decision was made to manage her reconstruction in a staged fashion, starting with debridement, removal of all nonviable bone, temporization of the resulting bone defect with antibiotic cement, preparation of the joint surfaces in anticipation of future tibiotalar fusion, and application of a multiplanar thin-wire circular external fixator (TWCEF). After infection clearance and soft tissue optimization, she returned to the operating room for definitive reconstruction. (**C**) To fill the bone void, a trough was burred into the talar dome, and then a fibular strut was fashioned to fit within the tibial canal and dock into the talar dome. The structural allograft was supplemented with cancellous autograft. (**D**) Compression was then applied across the TWECF, and a plate was applied for supplemental fixation.

Whether the result of initial bone loss at the time of injury, debridement, or deformity correction, bone defects are a complicating factor that must be navigated in revision reconstruction of pilon fractures. For smaller voids, bone grafting is indicated. Autogenous bone graft is the standard as it provides both osteoinductive and osteoconductive properties, maximizing the chances of healing in a hostile microenvironment [9,10,28,29]. The most common harvest sites for cancellous autograft are the anterior and posterior iliac crest, but alternate options include the femoral intramedullary canal via a reamer, irrigator aspirator (RIA), and, for smaller volumes, the proximal tibia [10,22,28]. Some bone defects require mechanical support, in which case the iliac crest cortical autograft can be utilized or, for larger structural voids, fibular strut or femoral head allograft [30,31]. Figure 1 details the case of a 24-year-old male who sustained a pilon fracture during a motor-vehicle collision. He presented six months after his initial fixation with an infected nonunion that had failed attempts at irrigation and debridement with hardware retention. He was managed in a staged fashion with an initial debridement resulting in a large metaphyseal defect that was temporized with antibiotic cement. After infection clearance, he underwent tibiotalar fusion using a combination of autologous cancellous autograft and structural femoral head allograft to fill the bone void and maintain the length of the extremity. Figure 2 displays another example of an infected nonunion treated in a staged fashion using a structural allograft, although in this case, a fibular strut is used to fill the critical bone defect.

**Figure 3:**
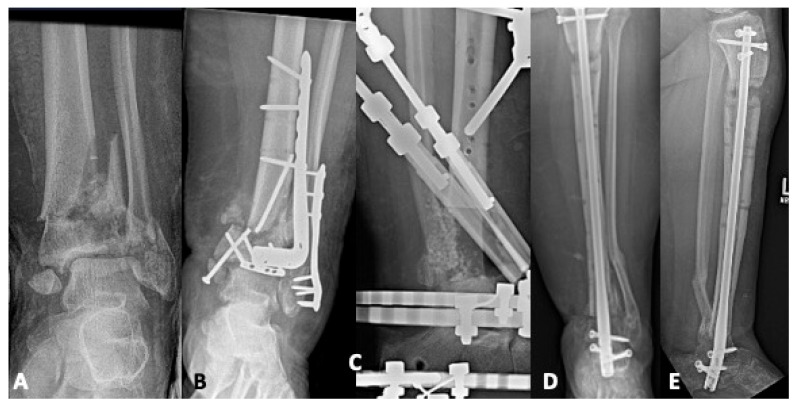
A thirty-five-year-old who sustained a Gustilo-Anderson Type II open pilon fracture in a motor vehicle collision. (**A**) Her initial injury was characterized by significant articular comminution and relative metaphyseal bone loss, and (**B**) her course was complicated by infected nonunion. At four months post-operative from her index procedure, she underwent removal of hardware and adequate debridement, leaving a roughly 5 cm bone defect. (**C**) To manage the void, a thin-wire circular external fixator was utilized to perform bone transport. (**D**,**E**) After 4 months, transport was complete, and the regenerate was mature. She underwent tibiotalocalcaneal arthrodesis for definitive treatment using an antibiotic-coated tibial nail inserted in a retrograde fashion through the calcaneus.

For bone deficits larger than 4 cm, a bone graft is insufficient to achieve healing due to necrosis and resorption [28,32]. The use of custom titanium cages has been reported, and personalized implants may be the future for management of defects in this category, but the current standard is distraction osteogenesis [9,22,33]. Methods utilizing distraction osteogenesis for critical bone defects can be grouped into two broad categories—bone transport and acute shortening combined with lengthening [25]. In the former, the tibia remains at its goal length through the procedure. An osteotomy is performed in the proximal tibia, and the resulting intercalary segment is moved toward the distal tibia at a rate of 0.5−1 mm per day and eventually docked into the distal fragment [34]. An additional procedure is often performed after the transport is complete to promote union at the docking site [35]. Bone transport has traditionally been accomplished with TWCEF, but plate-assisted transport over an intramedullary nail has also been performed [9,36]. Figure 3 demonstrates an example of TWCEF being used for bone transport to address a 5 cm bone defect in an infected pilon nonunion.

Shortening combined with lengthening is another alternative for non-graftable defects. In this technique, the distal bone void is resolved via acute shortening [35]. Separately, in the proximal tibia, a corticotomy is created, and the length lost distally in the shortening is regained through distraction osteogenesis at this site [9,25]. Proponents of this method note that it shifts the osteogenesis outside the zone of injury and, in some cases, prior infection [9]. Additionally, acute shortening removes the need for bone graft and may allow for primary closure of soft tissue defects, which can be particularly beneficial in patients who are poor candidates for free flaps [25]. The primary disadvantage to this approach is that a limited length of acute shortening can be tolerated by the vasculature supplying the foot [34]. Using either bone transport or acute shortening followed by distraction osteogenesis, conversion to an intramedullary nail after the distraction phase is completed can minimize the time a patient spends in TWCEF [37]. Photodynamic bone stabilization is a promising area of ongoing research that may offer an alternative to traditional intramedullary devices for regenerative stabilization during the consolidation phase [38]. It affords increased flexibility in the revision pilon setting where standard interlocking screws could be located in an area of soft tissue injury or prior infection [38].

### 4.3. Malunion

One potential consequence of failure to recognize and account for a bone defect during initial operative fixation of a pilon fracture is malunion. Malunion in pilon fractures can be divided into two overarching groups based on anatomic location—the metaphyseal region and the periarticular zone [11]. Metaphyseal malunions typically result from failure to appropriately manage a metadiaphyseal defect resulting from the initial injury [39]. This causes a deviation of the mechanical axis, most commonly in the coronal plane, although tibial recurvatum deformity can also occur [40]. A supramalleolar opening or closing wedge osteotomy can be utilized to correct varus or valgus malalignment [11]. This technique restores the symmetric distribution of forces across the ankle joint and can be a successful method for joint preservation in appropriately indicated patients [41]. Alternatively, a dome osteotomy can also address coronal deformity of the distal tibia [11,42,43]. Because a dome osteotomy does not occur through the center of rotation of angulation, it will induce translation, but has the benefits of reliable bone contact, which confers inherent stability without the need for bone graft or shortening [11,42,43].

Periarticular malunions are common and predispose patients to rapid progression of post-traumatic arthritis [11,44,45]. In cases of intra-articular malunion, a supramalleolar osteotomy is less effective at correcting alignment because it does not prevent the talus from continuing to collapse into the tibial plafond defect, driving the deformity [45]. An intra-articular osteotomy, also termed a “plafond-plasty,” has been described to address this issue by locating the osteotomy at the intra-articular step-off [46]. Li et al. describe a four-step framework for utilizing this technique specifically in the setting of revision fixation for malunion after pilon fracture: osteotomy for exposure of impacted articular fragments, articular surface reconstruction using a second osteotomy over the subchondral bone to achieve a congruent ankle joint, structural bone grafting, and osteotomy fixation [46]. Accurate reconstruction of the articular surface in a revision pilon can be particularly challenging, given the distorted anatomy resulting from displacement and remodeling. If the talar dome is intact, its contour can be utilized as a template for reduction in the distal tibia fragments to create a congruent ankle joint [46].

Finally, TWCEF is a powerful tool for addressing malunion of the distal tibia, especially in the context of multiplanar deformity, deformity with concurrent infection, or deformity that requires gradual correction [9,11]. It results in minimal soft tissue insult, avoids permanent hardware, and in some scenarios allows for earlier weight-bearing [9,11]. TWCEF is particularly suited to cases of malunion with minimal distal tibial bone stock but a preserved joint since it can achieve adequate stability for healing with a smaller footprint than plate and screw constructs [9]. Modern hexapod frames offer the additional advantage of executing a deformity correction plan specifically tailored to a patient’s individual deformity [11]. 3D-printed models and cutting guides for osteotomies offer customized options for other approaches to deformity correction, but TWCEF is unique in offering the opportunity for continued modification during the post-operative protocol based on patient progress [11,25].

## 5. Revision Surgical Options

### 5.1. Revision Operative Fixation

**Figure 4:**
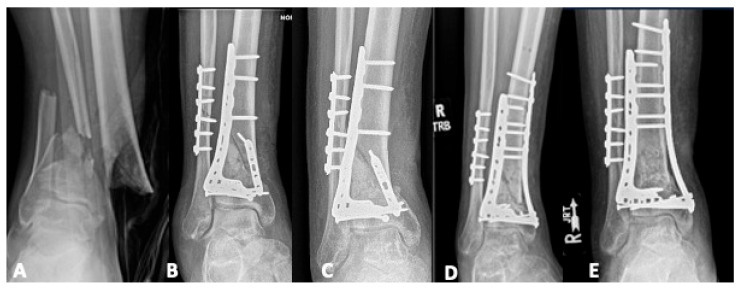
(**A**) A thirty-five-year-old man who sustained a Gustilo-Anderson type II open pilon fracture after falling 20 ft from a ladder. (**B**,**C**) His course was complicated by aseptic nonunion with progressive deformity. (**D**) He was taken back to the operating room five months after his original injury and procedure, where revision operative fixation was performed with the addition of a medial buttress plate to correct the varus deformity and an autologous bone graft to improve biology at the nonunion site. (**E**) One year after his revision, he was ambulating without assistance and had returned to work.

Arthrodesis was once thought to be the only viable option for limb salvage in a patient who failed initial pilon fixation [2]. However, a select population with certain patterns of malunion or nonunion and favorable patient characteristics may be appropriate candidates for isolated revision operative fixation. Indication criteria include a young, compliant, active patient with no infection, a healthy soft tissue envelope, minimal bone loss, and a largely preserved tibiotalar joint surface [9,47]. Although the minority of cases meet these criteria, supramalleolar axial deviation with a reduced joint surface is one scenario that can be amenable to a more limited intervention [9]. This deformity can occur with failure to appreciate and address the relative bone loss from comminution in the metaphyseal region during the index surgery [9,39,45]. Figure 4 displays the radiographs of a 35-year-old laborer who sustained a pilon fracture after a fall from a ladder. He developed an aseptic nonunion and progressive varus deformity that was treated with revision operative fixation and ultimately went on to union.

A variety of techniques and fixation options exist for correcting isolated deformity, as outlined in the malunion section above. Detailed pre-operative planning should include possible concomitant procedures that may be required to optimize alignment or function. Some of the more common adjunctive interventions include ligament reconstruction, Achilles release, cheilectomy of the talar neck, fibular osteotomy, calcaneus osteotomy, and peroneus longus to brevis transfer [11]. Finally, despite the goal of revision operative fixation, surgeons should also be prepared to perform tibiotalar arthrodesis. Pre-operative imaging may underestimate articular cartilage damage, and the ultimate decision to revise versus fuse should be made at the time of reconstructive surgery based on direct inspection [45].

### 5.2. Joint-Sacrificing Reconstruction

#### 5.2.1. Total Ankle Arthroplasty

The most common complication following a pilon fracture is post-traumatic arthritis. Even with meticulous surgical technique, almost all patients will show radiographic signs of osteoarthritis, and up to 39% have been reported to develop clinically symptomatic post-traumatic arthritis [2,48]. The surgical options to address a painful, arthritic ankle joint are total ankle arthroplasty (TAA) and tibiotalar arthrodesis. TAA preserves tibiotalar motion, decreasing the stress on adjacent joints and associated increased risk of arthrosis [49]. The preserved range of motion in TAA has been linked to improved gait mechanics and higher patient-reported outcomes than tibiotalar arthrodesis [50,51]. However, these data were collected from heterogeneous populations of patients with end-stage degenerative arthritis of the tibiotalar joint. Much less is known about TAA performance in patients with a history of pilon fracture. In a retrospective review of patients who underwent TAA for post-traumatic arthritis, Megerian et al. found a higher rate of complications and failure requiring prosthesis explant in patients with a history of a fracture [52]. However, a separate study found no significant difference in patient-reported outcomes for TAA performed for post-traumatic arthritis following pilon fracture as compared to those for patients undergoing TAA for osteoarthritis, rheumatoid arthritis, and ankle fracture [49].

**Figure 5:**
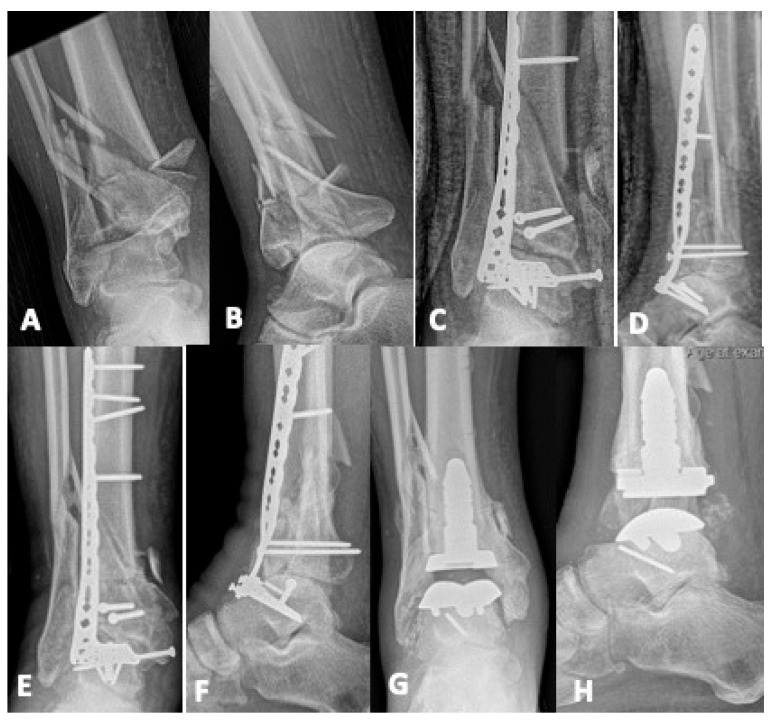
(**A**,**B**) A seventy-one-year-old who sustained a pilon fracture after falling off a retaining wall. (**C**,**D**) He was initially treated with acute tibiotalar fusion. (**E**,**F**) Nine months after his arthrodesis, he had continued pain that limited his daily life. Despite his chronological age, prior to his injury, he was very physically active on his farm and motivated to return to activity. The decision was made to perform a total ankle arthroplasty in a staged fashion with hardware removal, culture collection, and confirmation of the fracture union in the first phase. (**G**,**H**) He then underwent total ankle arthroplasty in the second stage.

While the outcomes of TAA in patients with a history of pilon fracture are an area that warrants additional investigation, the current indications remain narrow. Infection, critical bone defects, and coronal malalignment >10 degrees—common comorbid conditions with post-traumatic arthritis in the setting of the failed pilon—preclude total ankle arthroplasty and would need to be rectified prior to considering joint replacement [52,53]. The condition of the skin and underlying soft tissue is also of utmost importance, as infection and anterior wound-healing issues are known potential complications of TAA even in patients without a previous trauma. Finally, the location of incisions from the index surgery may also pose a problem if they cannot be used for TAA and prevent the placement of an appropriate new incision while maintaining a sufficient skin bridge. In our practice, the optimal candidate for TAA following failed pilon fixation has symptomatic tibiotalar arthritis with no evidence of infection, an amenable soft tissue envelope, and minimal to no deformity. Ideally, the patient is active enough to benefit from the preserved tibiotalar motion but not so high-demand that the durability of the implant becomes an issue. Figure 5 demonstrates one such patient, a 71-year-old farmer who sustained a pilon fracture after falling off a retaining wall. He was initially managed with attempted acute tibiotalar fusion, but at nine months, he had persistent pain that limited his activity. His revision surgery was performed in a staged fashion with hardware removal and culture collection to confirm the absence of infection, followed by a total ankle arthroplasty.

#### 5.2.2. Tibiotalar Arthrodesis

The workhorse of revision pilon fixation is tibiotalar arthrodesis. It is the most employed surgical strategy for limb salvage following failed pilon operative fixation and has been demonstrated to successfully relieve pain, correct malalignment, and restore function [54,55]. A wide variety of techniques have been described to accomplish tibiotalar arthrodesis, including fixation with plates and screws, intramedullary nailing, and TWCEF [9,10,22,27,56]. Each approach has a distinct risk-benefit profile that should be considered relative to the patient and nonunion characteristics when deciding on an operative plan. Compared to intramedullary devices, plates offer increased options for fixation, especially in the distal segment, and have demonstrated higher stiffness in biomechanical studies [57]. However, they require a more invasive, open approach and can be more technically challenging to use, especially with blade plates [58]. Intramedullary devices are minimally invasive, but tibiotalarcalcaneal nails sacrifice the subtalar joint, and antegrade nailing with a long tibial nail is not optimized for fixation distal to the tibiotalar joint [27,56,59].

As we have emphasized previously, TWCEF frames are versatile tools for addressing a variety of goals in the revision pilon setting, and this extends to performing tibiotalar arthrodesis. Their ability to simultaneously lengthen the tibia and compress across the tibiotalar joint deserves specific attention [9,22]. In the setting of infection, an appropriately aggressive debridement leaves a bone defect that must be reconstructed. Even in cases of aseptic nonunion, defects may result from deformity correction or excision of nonviable bone [1,9]. TWCEF can be constructed in such a way as to allow distraction osteogenesis through a proximal corticotomy while simultaneously compressing across the tibiotalar joint, solving both problems with one device [22,25]. The authors’ preferred method for constructing a bifocal frame to achieve concurrent lengthening and tibiotalar arthrodesis starts with placing two rings in the proximal tibia with two wires per ring to achieve multiplanar fixation. The most proximal of these rings can be a 2/3 ring to allow for increased knee range of motion. The segment of the tibia from the corticotomy to the distal end of the tibia is controlled with two full rings, each with two wires per ring. Half-pins can be added for additional fixation, or they can be used to replace wires where appropriate. The distal ring is placed as close to the distal tibia as possible to increase stability during tibiotalar compression. Wires are placed through the calcaneus and talus on a footplate and connected to the intercalary segment. This construct offers several dimensions of flexibility—the rate of lengthening can differ from the rate of compression, small adjustments in coronal or sagittal alignment can be made via the post-operative protocol instead of requiring revision surgery, and any acute shortening required to improve bony contact or support soft tissue closure at the time of frame application can be compensated for in the distraction osteogenesis plan [25].

**Figure 6:**
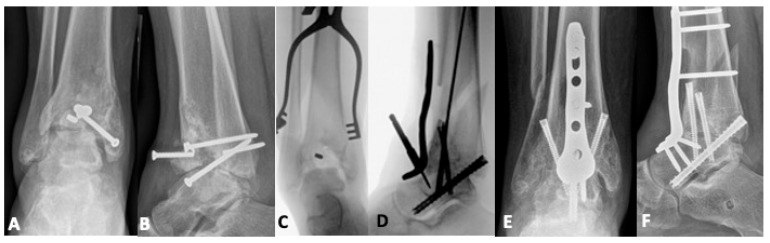
(**A**,**B**) A twenty-nine-year-old who presented to our institution six months after a pilon fracture with attempted internal fixation complicated by nonunion and malalignment. (**C**) After debridement of a substantial amount of nonviable bone, she had an anterior defect of approximately 2 cm. (**D**) The decision was made to proceed with a modified Blair arthrodesis, sliding a piece of the anterior piece of the tibia cortex distally and docking it into a trough crated in the talar neck. (**E**,**F**) Four months after her arthrodesis, she was pain-free and beginning to weight-bear.

TWCEF is not a viable solution for all patients. It is a resource-intensive intervention characterized by a prolonged treatment course and expected complications. As such, it requires a compliant and engaged patient with social support [9,25]. TWCEF frames are also not equally suited for certain malunion/nonunion patterns. Arthrodesis of the tibia to the hindfoot may be more difficult to achieve in the setting of concurrent talus pathology due to less bone stock availability and less stability in the TWCEF [22]. For patients undergoing fusion following a pilon fracture complicated by a septic nonunion involving the ankle joint or damage to the talar body at the time of the original injury, the Blair fusion can be an alternative option. Originally described as an option to address degenerative changes in the tibiotalar joint following talus fractures, the technique utilizes a corticocancellous graft from the anterior tibia to connect the tibial shaft to the talar neck [60]. Figure 6 demonstrates the authors’ preferred modification of the originally described technique, which incorporates an anterior plate and cannulated headless compression screws to provide added stability.

### 5.3. Amputation

A review of options for treatment of pilon fractures that have failed prior operative intervention would be incomplete without addressing amputation. Pilon fractures are life-changing injuries, and limb salvage is not always possible or prudent [7]. Several studies have demonstrated similar long-term outcomes and high complication rates when comparing limb salvage and amputation in high-energy tibial fractures [5,6,61,62]. An elective amputation is a deeply personal decision, and satisfaction with a minimally functional reconstruction versus a prosthesis is influenced by a myriad of psychosocial factors [9]. The optimal strategy for complex lower extremity injuries will vary and is best developed through shared decision-making with the patient. However, it is incumbent upon the surgeon to understand the existing literature and present amputation as a treatment option and not a failure or last resort.

## 6. Conclusions

Pilon fractures failing initial management span a spectrum of severity from isolated varus collapse to limb-threatening infected nonunion with a critical bone defect. Patients also vary widely in both their baseline host status and their goals of care. No single strategy can be aptly applied to such a broad range of pathology, but this review outlines a reproducible, prioritized framework that can be adapted to individual cases—eradicate infection, restore mechanical stability, correct deformity, and augment biological healing potential. These tenets can be incorporated into a personalized joint-preserving or joint-sacrificing reconstruction plan based on host factors and functional goals. The management of failed pilon fractures is a promising field for future research, given the current limited and largely retrospective literature. Further advances in the care of these complex injuries will depend on patient-centered treatment plans that combine higher-quality evidence with mechanical and biological innovation.

## Data Availability

No new data were created or analyzed in this study. Data sharing is not applicable to this article.
